# Meeting the global need for physician-scientists: a Middle Eastern imperative

**DOI:** 10.3402/meo.v19.26138

**Published:** 2014-10-30

**Authors:** Lucman A. Anwer, Ayesha N. Anwer, Maryam Mahmood, Ahmed Abu-Zaid, Mohammad Abrar Shareef

**Affiliations:** 1 College of Medicine, Alfaisal University, Riyadh, Saudi Arabia; 2 College of Dentistry, CMH Lahore Medical College & Institute of Dentistry, Lahore, Pakistan

The plight and importance of physician-scientists has been discussed repeatedly over the last few years (1, 2). Their ability to incorporate bench research with clinical practice makes them a unique species – perfect for pioneering the field of translational research (3, 4).

A gradual realization of their significance has occurred in the West, with more students enrolling into MD-PhD programs. Unfortunately, there is a theoretical limit as to how much raw material can be fed into the system to produce the desired product. Therefore, to ensure that the net supply of MD-PhDs remains on the rise rather than reaching equilibrium, an ‘alternate’ source of trainees is needed.

Unlike the West, where MD-PhD programs have been a vital force in increasing physician-scientists, such trends have yet to be seen elsewhere – specifically, in the Middle East (5). Examining root causes of this situation beckons two questions: 1) Are medical students sufficiently motivated to engage in the field of research? and 2) Is the infrastructure available to support the process?

The first of these two questions revolves around suboptimal early research exposure. Recent scholars have discussed this problem in-depth, and modest but positive efforts have already begun to rectify this situation (6). However, we believe that it is the second of these two questions which forms the gist of the dilemma.

Students in the Middle East have yet to enjoy the option of applying to formal MD-PhD programs – leaving research-oriented medical students no choice but to attain a PhD following their MD. While this strategy may suffice for those physician-scientists planning solely on research careers, it is far less ideal for those wishing to practice both medicine and research.

First and foremost among the barriers is the prolonged duration of ‘stand-alone’ PhD programs, which, in the United States, takes a minimum of 3.5–4 years to complete. This leads to major time off from clinical practice – a risk not everyone is willing to take, since 3–4 years of laboratory work can lead to a stagnation of one's clinical skills.

Second, but perhaps more importantly, are the financial considerations of embarking on a PhD. According to 2011 data from the Association of American Medical Colleges (AAMC), a U.S. medical resident earns, on average, $49,000 annually; in contrast, the average stipend of a PhD student is roughly half this figure (7). Enduring this substantive salary differential for 3.5–4 years is simply not a viable option for many MDs.

The growing need for physician-scientists calls for Middle Eastern governments and education-governing bodies to invest in formal, joint MD-PhD, residency-PhD, and fellowship-PhD programs. Tapping into the human potential of this region is one step toward easing the global demand for clinical scientists. The Middle East has long served the world as a ‘black gold’ reservoir; we believe it's time that it offers more.

*Lucman A. Anwer* College of Medicine Alfaisal University Riyadh Saudi Arabia
Email: lucman_anwer@hotmail.com *Ayesha N. Anwer* College of Medicine Alfaisal University Riyadh Saudi Arabia *Maryam Mahmood* College of Dentistry CMH Lahore Medical College & Institute of Dentistry Lahore Pakistan *Ahmed Abu-Zaid* College of Medicine Alfaisal University Riyadh Saudi Arabia *Mohammad Abrar Shareef* College of Medicine Alfaisal University Riyadh Saudi Arabia
